# Negative effects of a high tumour necrosis factor-α concentration on human gingival mesenchymal stem cell trophism: the use of natural compounds as modulatory agents

**DOI:** 10.1186/s13287-018-0880-7

**Published:** 2018-05-11

**Authors:** Chiara Giacomelli, Letizia Natali, Marco Nisi, Marinella De Leo, Simona Daniele, Barbara Costa, Filippo Graziani, Mario Gabriele, Alessandra Braca, M. Letizia Trincavelli, Claudia Martini

**Affiliations:** 1 Department of Pharmacy, University of Pisa, Via Bonanno 6, 56126 Pisa, Italy; 2 Department of Surgical, Medical, Molecular and Critical Area Pathology, University of Pisa, Via Savi 10, 56126 Pisa, Italy; 3 Centro Interdipartimentale di Ricerca “Nutraceutica e Alimentazione per la Salute”, University of Pisa, Via del Borghetto 80, 56124 Pisa, Italy

**Keywords:** Human gingival mesenchymal stem cells, Tumour necrosis factor-alpha, Endothelial cells, Cytokine release, *Ribes nigrum*

## Abstract

**Background:**

Adult mesenchymal stem cells (MSCs) play a crucial role in the maintenance of tissue homeostasis and in regenerative processes. Among the different MSC types, the gingiva-derived mesenchymal stem cells (GMSCs) have arisen as a promising tool to promote the repair of damaged tissues secreting trophic mediators that affect different types of cells involved in regenerative processes. Tumour necrosis factor (TNF)-α is one of the key mediators of inflammation that could affect tissue regenerative processes and modify the MSC properties in in-vitro applications. To date, no data have been reported on the effects of TNF-α on GMSC trophic activities and how its modulation with anti-inflammatory agents from natural sources could modulate the GMSC properties.

**Methods:**

GMSCs were isolated and characterized from healthy subjects. The effects of TNF-α were evaluated on GMSCs and on the well-being of endothelial cells. The secretion of cytokines was measured and related to the modification of GMSC-endothelial cell communication using a conditioned-medium method. The ability to modify the inflammatory response was evaluated in the presence of *Ribes nigrum* bud extract (RBE).

**Results:**

TNF-α differently affected GMSC proliferation and the expression of inflammatory-related proteins (interleukin (IL)-6, IL-10, transforming growth factor (TGF)-β, and cyclooxygenase (COX)-2) dependent on its concentration. A high TNF-α concentration decreased the GMSC viability and impaired the positive cross-talk between GMSCs and endothelial cells, probably by enhancing the amount of pro-inflammatory cytokines in the GMSC secretome. RBE restored the beneficial effects of GMSCs on endothelial viability and motility under inflammatory conditions.

**Conclusions:**

A high TNF-α concentration decreased the well-being of GMSCs, modifying their trophic activities and decreasing endothelial cell healing. These data highlight the importance of controlling TNF-α concentrations to maintain the trophic activity of GMSCs. Furthermore, the use of natural anti-inflammatory agents restored the regenerative properties of GMSCs on endothelial cells, opening the way to the use and development of natural extracts in wound healing, periodontal regeneration, and tissue-engineering applications that use MSCs.

**Electronic supplementary material:**

The online version of this article (10.1186/s13287-018-0880-7) contains supplementary material, which is available to authorized users.

## Background

Tissue regeneration is a dynamic process divided into different organized stages: haemostasis, an inflammatory phase, proliferation, and maturation/matrix remodelling [1, 2]. All these phases require cell-to-cell interactions orchestrated by growth factors, cytokines, and extracellular matrix (ECM) components [3], and a dysregulation of these mediators could lead to a pathological wound healing and fibrosis [4]. Among the different phases, several cell types are involved including mesenchymal stem cells (MSCs), endothelial cells, immune cells, and fibroblasts. During the initial stages, plenty of pro-inflammatory factors are released by resident or immune cells attracted to the site of injury [5] that play a role in the activation of MSC-driven tissue repair and angiogenesis. Among these inflammatory factors, tumour necrosis factor (TNF)-α is of great interest for its ability to influence the properties of MSCs [6, 7], varying the secretion of soluble mediators that could interfere with endothelial functionality and tissue regeneration [8]. TNF-α is considered a pleiotropic cytokine with diverse effects that range from triggering proliferation to inducing apoptosis, dependent on cell type, status, environment, time, and dose of exposure [9, 10]. Thus, the effects of different concentrations of TNF-α on the gingiva-derived MSC (GMSC) subtype could be of great interest for scientists in the field of regenerative medicine.

Stem cell biology has become an important field in regenerative medicine and tissue engineering therapy since the discovery and characterization of MSCs. MSCs represent a population of multipotent stem cells that can be isolated from many tissues [11, 12]. The isolation of MSCs produces heterogeneous, non-clonal cultures of stromal cells containing stem cells with different multipotential properties, committed progenitors, and differentiated cells [13]. However, cultures obtained from bone marrow (BM) and other tissues that contain a subpopulation of stem cells are currently serving as a valuable source of accepted MSCs for therapeutic purposes [14, 15]. Among the different population of stem cells, GMSCs have attracted great attention due to their easy accessibility from the oral cavity [16, 17]. GMSCs are stem cells derived from the neuronal crest; besides the well-established self-renewal, multipotent differentiation, and immunomodulatory properties, GMSCs, similar to other MSCs, possess trophic activity which could be of great therapeutic interest [18]. Gingival tissues also exhibit scarless wound healing properties and a high regenerative capability, highlighting these cells as an attractive therapeutic option to enhance wound repair in oral and extra-oral tissues [19, 20].

Despite efforts towards the discovery of agents and mechanisms that could modulate and promote regeneration, to date several issues remain to be resolved. In fact, the development of an adequate vascular network remains one of the major challenges in tissue regeneration [21]. Although tissue engineering has provided scaffolds that are widely used in clinics for skin wound healing [22] and periodontal regeneration [23, 24], the limitation of oxygen diffusion remains a key issue. Thus, improvement of angiogenic processes to provide adequate blood supply for cells under proliferation could represent a pivotal milestone. In this scenario, the use of natural products that are able to directly improve the functions of the endothelial cells or to modulate the cell microenvironment has become as a promising tool.

Over the last decade, much research has highlighted the beneficial role of fruit and vegetable consumption in lowering the risk for developing chronic inflammation [25, 26]. The *Ribes nigrum* L. (blackcurrant) is a small, perennial shrub that belongs to the family Grossulariaceae. The *R. nigrum* bud extract (RBE) contain vitamins, terpenic, and phenolic compounds, including flavonols, phenolic acids, and catechins at high concentrations [27, 28]. The blackcurrant has been shown to exhibit several biological properties, such as anti-microbial, anti-oxidant and anti-inflammatory activities [29]. Interestingly, the in-vitro administration of a berry and leaf extract is able to contrast the effects of TNF-α and to modulate the cytokine release of monocytes [30]. The ability to modulate inflammatory pathologies and the positive effects against dermal diseases (eczema and psoriasis) [29, 31] shows the potential effect of the extract in the regeneration of injured tissues.

To date, no data have been reported on the effects of TNF-α on GMSC trophic properties and how its modulation with anti-inflammatory agents from natural sources could restore the GMSC functions. Thus, the aim of this work was to investigate the effects of TNF-α on the well-being of GMSCs and on the GMSC/endothelial cell interplay. Furthermore, the possibility of using a natural extract (RBE) to restore the physiological trophic properties of GMSCs was evaluated.

TNF-α differently affected the GMSC proliferation and expression of inflammatory-related proteins dependent on its concentration. A high TNF-α concentration produced an increase in pro-inflammatory proteins, reducing the positive effects of the GMSC secretome on endothelial cells. RBE, which was rich in phenol constituents with anti-inflammatory activity, was able to affect the GMSC release of inflammatory mediators, thus restoring endothelial cell migration and healing under physiological and pathological conditions.

## Methods

### Materials

A hydro-alcoholic glycerine solution of *R. nigrum* buds (1.5%) was kindly provided by Biokyma S.r.l. (Anghiari, Arezzo, Italy). The RNeasy® Mini Kit was obtained from Qiagen S.p.A. The iScript cDNA synthesis kit was purchased from Bio-rad s.r.l. Fluocycle® II SYBR® was purchased from Euroclone s.p.a. (Milan, Italy). TNF-α was purchased from Sigma Aldrich (Milan, Italy). High-performance liquid chromatography (HPLC)-grade water (18 mΩ) was prepared by a Mill-Ω^50^ purification system (Millipore Corp., Bedford, MA, USA). All the reagents and materials were obtained from commercial sources with a high grade of purity.

### Isolation and culture of human GMSCs

GMSCs were obtained after processing de-keratinized gingival tissues previously collected from four healthy female patients (average age 35.5 years) undergoing clinical crown lengthening procedures. The protocol received approval from the ethical committee of the University Hospital of Pisa (Pisa, Italy; protocol no. 32835/2016) and informed consent was obtained from the included patients.

The tissues were processed as previously reported with a few modifications [32]. Briefly, after surgical removal, discharged gingival specimens were de-epithelialized and placed in sterile phosphate-buffered saline (PBS) with 100 U/mL penicillin and 100 μg/mL streptomycin (Sigma-Aldrich, Milan, Italy) at 4 °C. The tissues were minced into 1–2 mm^2^ fragments and digested in Dulbecco’s modified Eagle’s medium (DMEM)-F12 containing dispase (1 mg/mL; Sigma-Aldrich) and collagenase IV (2 mg/mL; Sigma-Aldrich) at 37 °C for 30 min. Then, the suspension was discarded, and the remained tissues were digested in the same solution for 90 min at 37 °C. The solution was then filtered with a 70-μm cell strainer (Sigma-Aldrich) and seeded with DMEM-F12 containing 10% fetal bovine serum (FBS), 100 U/mL penicillin, 100 μg/mL streptomycin, and 200 mM l-glutamine in a 25-cm^2^ tissue culture flask. At 24 h after isolation, the non-adherent cells were washed with PBS and replaced with fresh medium (passage 0).

### Cell cultures

The isolated GMSCs were maintained in growth medium (DMEM-F12 containing 10% FBS, 100 U/mL penicillin, 100 μg/mL streptomycin, 200 mM l-glutamine) and incubated at 37 °C in 5% CO_2_ and 95% air. The medium was changed to remove non-adherent cells every 3 to 4 days and the cells were used at passages 0 to 6.

Human dermal fibroblasts cells (HuDe; purchased from the Istituto Zooprofilattico Sperimentale, Brescia, Italy) were maintained in DMEM-F12 containing 10% FBS, 100 U/mL penicillin, 100 μg/mL streptomycin, and 200 mM l-glutamine, and incubated at 37 °C in 5% CO_2_ and 95% air. The medium was changed to remove non-adherent cells every 3 to 4 days and the cells were used at passages 0 to 20.

The immortalized human microvascular endothelial cell line (HMEC-1) was obtained from ATCC and maintained in a culture medium of MCDB131 (without l-glutamine), 10 ng/mL epidermal growth factor (EGF), 10 μg/mL hydrocortisone, 10 mM l-glutamine, and 10% FBS. Cells were maintained in a 37 °C humidified incubator with 5% CO_2._ The medium was changed to remove non-adherent cells every 3 to 4 days and the cells were used at passages 10 to 18.

### Colony-forming unit-fibroblast (CFU-F) assay and doubling time

For CFU-F assay, the GMSCs were plated into a six-well plate at densities of 500 or 1000 cell/well and maintained in growth medium. After 14 days, the cells were fixed with 4% paraformaldehyde (Sigma-Aldrich) and stained with 1% crystal violet. Groups of ≥ 50 cells were scored as a CFU-F colony. The numbers of colonies were statistically evaluated.

The assessment of the GMSC doubling time was performed as previously described [33]. Cells of different passages were seeded onto 12-well plates at 10^3^ cells/well in growth media and the cells in each well were scored at 24, 48, 72, and 96 h. Population doubling was calculated using: number of divisions = log2 (number of cells at subculture/number of cells seeded).

### PCR and real-time RT-PCR analysis

GMSC surface markers were analysed with polymerase chain reaction (PCR). The PCR reaction was conducted following the manufacturer’s instructions (GoTaq G2 Flexi DNA Polymerase, Promega) and with the following reagents: 5 μL GoTaq Flexi Buffer, 1.5 μL MgCl_2_ (25 mM), 0.5 μL PCR Nucleotide Mix (10 mM), 0.5 μL of forward and reverse primers (10 μM; Table 1), 0.2 μL GoTaq G2 Flexi DNA Polymerase (5 U/μL), 500 ng cDNA, and 11.8 μL H_2_O. The reactions were performed for 40 cycles using the following temperature profiles: 95 °C for 2 min; 55 °C for 30 s, and 72 °C for 5 min. β-actin was used as the housekeeping gene.

**Table 1:** Primers used for real-time RT-PCR

Gene	Primer nucleotide sequences	Product size (base pairs)	Annealing temperature
CD34	Forward: 5’-GCCTGGAGCAAAATAAGACCTC-3’ Reverse: 5’-AGGATCCCCAGCTTTTTCAGG-3′	250 bp	55 °C
CD45	Forward: 5’-TGTGGAGCCAATCCATGCAGA-3’ Reverse: 5’-GTTTGACCCTGCATCTCCGTT-3′	232 bp	55 °C
CD90	Forward: 5’-CACACATACCGCTCCCGAA-3’ Reverse: 5’-CACCAGTCACAGGGACATGAA-3′	279 bp	55 °C
CD105	Forward: 5’-ATACCACTAGCCAGGTCTCGAA-3’ Reverse: 5’-ATGGCAGCTCTGTGGTGTTG-3′	288 bp	55 °C
NF-kB	Forward: 5′-CAGCAGATGGCCCATACCTT-3′ Reverse: 5′-CACCATGTCCTTGGGTCCAG-3′	287 bp	55 °C
mTOR	Forward: 5′-GCCCCTACATGGAGCCTATTC-3′ Reverse: 5′-CCTGGAGCATGTCCATGATGA-3′	183 bp	55 °C
Oct4	Forward: 5’-CTCACCCTGGGGGTTCTATT-3’ Reverse: 5’-CTCCAGGTTGCCTCTCACTC-3’	230 bp	55 °C
SOX2	Forward: 5′-CATGAAGGAGCACCCGGATT-3′ Reverse: 5′-ATGTGCGCGTAACTGTCCAT-3′	186 bp	55 °C
TNF-α	Forward: 5′-AGGGACCTCTCTCTAATCAGCC-3′ Reverse: 5′-GCTTGAGGGTTTGCTACAACA-3′	101 bp	55 °C
IL-6	Forward: 5′-TCCTCGACGGCATCTCA-3′ Reverse: 5′-TTTTCACCAGGCAAGTCTCCT-3′	165 bp	55 °C
COX-2	Forward: 5′-TGTGTTGACATCCAGATCACAT-3′ Reverse: 5′-GGAGTCGGGCAATCATCAGG-3′	237 bp	55 °C
IL-10	Forward: 5′-CAAGCTGAGAACCAAGACCC-3′ Reverse: 5′-AAGATGTCAAACTCACTCATGGC-3′	141 bp	55 °C
TGF-β	Forward: 5′-ACTGCAAGTGGACATCAACG-3′ Reverse: 5′-TGCGGAAGTCAATGTACAGC-3′	218 bp	55 °C
β-actin	Forward: 5’-GCACTCTTCCAGCCTTCCTTCC-3’ Reverse: 5’-GAGCCGCCGATCCACACG-3’	254 bp	55 °C

The gene expression of GMSCs and HMECs were quantified by performing a real-time reverse transcription (RT)-PCR analysis. Briefly, GMSCs (5.0 × 10^3^ cell/cm^2^) or HMECs (1.0 × 10^4^ cell/cm^2^) were treated with glycerine-ethanol solution or RBE (50 μg/mL) in the absence or the presence of TNF-α (1 ng/mL or 10 ng/mL) for the indicated times. The cells were collected, and the total RNA was extracted using the Rneasy® Mini Kit (Qiagen, Hilden, Germany) according to the manufacturer’s instructions. The purity of the RNA samples was determined by measuring the absorbance at 260/280 nm. cDNA synthesis was then performed with 500 ng RNA using the i-Script cDNA synthesis kit following the manufacturer’s instructions. Real-time RT-PCR reactions consisted of 25 μL Fluocycle® II SYBR®, 1.5 μL of both 10 μM forward and reverse primers, 3 μL cDNA, and 19 μL H_2_O. All reactions were performed for 40 cycles using the following temperature profiles: 98 °C for 30 s; 55 °C for 30 s; and 72 °C for 3 s. The primers used were designed to span intron/exon boundaries and β-actin was used as the housekeeping gene. The mRNA levels for each sample were normalized against β-actin mRNA levels, and the relative expression was calculated using a Ct value. PCR specificity was determined by both melting curve analysis and gel electrophoresis.

### Immunofluorescence analysis of stem cell markers

GMSCs were seeded in an eight-chamber slide at a density of 5 × 10^3^ cells/chamber and incubated overnight. The next day, the cells were fixed with 2% paraformaldehyde (Sigma-Aldrich), and then permeabilized in 1% Triton- X100 with PBS for 3 min. GMSCs were stained with anti-CD90 (Millipore, cat. no. CBL415) and Alexa-Fluor-488-conjugated goat anti-mouse. Non-specific binding was controlled by omitting the primary antibody or by substituting the same concentration of non-specific isotype immunoglobulin (data not shown). Nuclei were stained with DAPI (1:5000 dilution; Sigma-Aldrich). The specimens were mounted in VECTASHIELD_ Mounting Medium with DAPI (Vector laboratories) and then observed under a fluorescence microscope (Axiovert 200; Zeiss).

### Western blot analysis

The protein expression of the surface marker CD90 was detected in GMSCs by performing a Western blot analysis. Cells were lysed for 60 min at 4 °C by the addition of 200 μL RIPA buffer. Equal amounts of the cell extracts (35 μg of proteins) were diluted in a Laemmli solution, resolved by SDS-PAGE (15%), and then transferred to PVDF membranes and probed overnight at 4 °C with primary antibody anti-CD90 (diluted 1:100; Millipore, cat no. CBL415) or β-actin (diluted 1:1000; MAB1501, Merck KGaA, Darmstadt, Germany). The primary antibodies were then detected using anti-rabbit IgG light chains conjugated to peroxidase (diluted 1:5000; 12–348; Millipore). The peroxidase was detected using a chemiluminescent substrate (ECL, Perkin Elmer), and the images were acquired by photographic film or by LAS4010 (GE Health Care Europe, Uppsala, Sweden). Immunoreactive bands were quantified by performing a densitometric analysis with Image J Software (version 1.41; Bethesda, MD, USA).

### Mineralization assay

GMSCs were seeded (3 × 10^3^ cells/cm^2^) in growth media. After 24 h the media was replaced with osteogenic-induction medium (Euroclone) containing dexamethasone (10^−8^ M), l-glutamine, ascorbate (50 μg/mL), penicillin/streptomycin (2 mM), and β-glycerophosphate (2 mM). As controls, GMSCs were cultured in growth medium. The medium was changed every 3 days and the mineralization was quantified after 15 or 21 days of treatment. The rate of mineralization was quantified using Alizarin Red staining as previously reported [34] with a few modifications. Briefly, cells were washed with PBS, fixed (4% formaldehyde in PBS) for 15 min, and then cells were stained with Alizarin Red S (1:100 in distilled water, adjusted to pH 4.2, and filtered), washed (five times) in 50% ethanol, and air dried. For quantification, cells were destained overnight in 10% (w/v) cetylpyridinium chloride at room temperature with continuous agitation and the absorbance (562 nm) was read using a spectrophotometer (Victor Wallac 2, Perkin Elmer).

### Cell viability assays

GMSCs and HMEC-1 were seeded in 96-well microplates (3.0 × 10^3^ cells/well) and treated with different concentrations of RBE (100 ng/mL to 100 μg/mL) or TNF-α (1–100 ng/mL) alone or in combination for 24, 48, or 72 h. Following the treatment period, cell proliferation was determined using an MTS assay (CellTiter 96 AQueous One Solution Cell Proliferation Assay kit; Promega) according to the manufacturer’s instructions. The absorbance of formazan at 490 nm was measured in a colorimetric assay with an automated plate reader (Victor Wallac 2, Perkin Elmer).

The cytotoxicity was assessed by a neutral red assay. Briefly, cells treated as above were incubated for 1 h with 100 μL of neutral red dye (33 μg/mL) dissolved in serum-free medium. At the end of the incubation period, cells were washed twice with the warm PBS solution. The cell-incorporated dye was solubilized in 100 μL of neutral red assay solubilization solution (50% EtOH and 1% AcOH). The cultures were allowed to stand for 10 min at room temperature with gentle shaking. The absorbance was measured at a wavelength of 540 nm. The background absorbance of multiwell plates at 690 nm was subtracted from the measurement at 540 nm.

### Analysis of apoptosis

For apoptosis measurements, GMSCs and HMEC-1 (5.0 × 10^3^ cell/cm^2^) were treated with or without TNF-α (100 ng/mL) for 72 h. The percentages of living, apoptotic, and dead cells were then quantified and analysed by the Muse™ Cell Analyzer (Merck KGaA, Darmstadt, Germany). The live, early apoptotic, and late apoptotic/dead cells were discriminated between using staining with Annexin V and 7-aminoactinomycin D (7-AAD).

### The protein-protein interaction networks (PPIN)

Network analysis was performed using the STRING (Search Tool for the Retrieval of Interacting Genes/Proteins) website (http://string-db.org/). The co-mentions, co-expression, and associations were set as the evidence for functional links with a medium confidence score of 0.4.

### Cytokine release

GMSCs were treated with glycerine-ethanol solution (control) or RBE (50 μg/mL) in the absence or the presence of TNF-α (1 ng/mL or 10 ng/mL) for 24 h. The amounts of cytokines presented in the culture medium (interleukin (IL)-6, IL-10, and transforming growth factor (TGF)-β) or expressed on the cell membrane (cyclooxygenase (COX)-2) was measured using enzyme-linked immunosorbent assay (ELISA) kits (Thermo Fisher Scientific, Rodano, Milan, Italy, Enzo Lifescience) following the manufacturers’ instructions.

### Wound healing analysis

GMSCs (5.0 × 10^3^ cell/cm^2^) were treated with glycerine-ethanol solution or RBE (50 μg/mL) in the absence or the presence of TNF-α (1 ng/mL or 10 ng/mL) for 24 h*.* Media from GMSCs were collected, and conditioned media in the proportion of 20% + 80% (20% GMSC conditioned media + 80% HMEC-1 culture media) were applied onto HMEC-1.

HMEC-1 were seeded in 96-well plates and grown to 90% confluence. A scratch was then made through the cell layer using a sterile micropipette tip. After washing with PBS, cells were treated with condition media from GMSCs treated as above. The images of the wounded area were captured immediately after the scratch (t_0_) and 8 h later (t_8_) to monitor cell regeneration into the wounded area. Photographs were then taken at 10× magnification on an inverted microscope. The wound-healing abilities were quantified by measuring both the average gap width and the percentage of gap closed. The data were analysed with Image J software.

### HPLC-PDA/UV-ESI-MS/MS analysis

For the HPLC-photodiode array (PDA)/UV-electrospray ionization (ESI) - tandem mass spectrometry (MS/MS) (HPLC-PDA/UV-ESI-MS/MS) study, 500 μL of a hydro-alcoholic glycerine solution of *R. nigrum* buds was added to 1.5 mL of methanol and the mixture was first centrifuged, then filtered and injected into the LC-MS system. Qualitative HPLC-PDA/UV-ESI-MS/MS analysis was performed using a Surveyor LC pump, a Surveyor autosampler, coupled with a Surveyor PDA detector, and a LCQ Advantage ion trap mass spectrometer (ThermoFinnigan, San Jose, CA, USA) equipped with Xcalibur 3.1 software. Analysis was performed using a 4.6 × 250 mm, 4-μm, Synergi POLAR-RP 80A column (Phenomenex, Castel Maggiore, Bologna, Italy). The eluent was a mixture of methanol (solvent A) and a 0.1% aqueous solution of formic acid (solvent B). The solvent gradient was as follows: 0–5 min, 5–50%; 5–25 min, 50% A isocratic mode; 25–85 min, 50–100% A. Elution was performed at a flow rate of 0.8 mL/min with a splitting system of 2:8 to MS detector (160 μL/min) and PDA detector (640 μL/min), respectively. The volume of the injected methanol solution was 20 μL. Analyses were performed with an ESI interface in the negative mode. The ionization parameters used were as follows: capillary temperature, 270 °C; capillary voltage, −16.0 V; tube lens offset, −5 V; sheath gas flow rate, 60.00 arbitrary units; auxiliary gas flow rate, 3.00 arbitrary units; spray voltage, 4.50 kV; scan range of *m/z* 150–1200 [35]. N_2_ was used as the sheath and auxiliary gas. PDA data were recorded in the 200–600 nm range, with preferential channels 254, 280, and 325 nm as the detection wavelengths.

### Statistical analysis

The GraphPad Prism (GraphPad Software Inc., San Diego, CA, USA) was used for data analysis and graphical presentations. All data are presented as the mean ± SEM. Statistical analysis were performed by a one-way analysis of variance (ANOVA) with Bonferroni’s corrected *t* test for post-hoc pair-wise comparisons. *P* < 0.05 was considered as statistically significant.

## Results

### Characterization of the isolated GMSCs

**Fig. 1:**
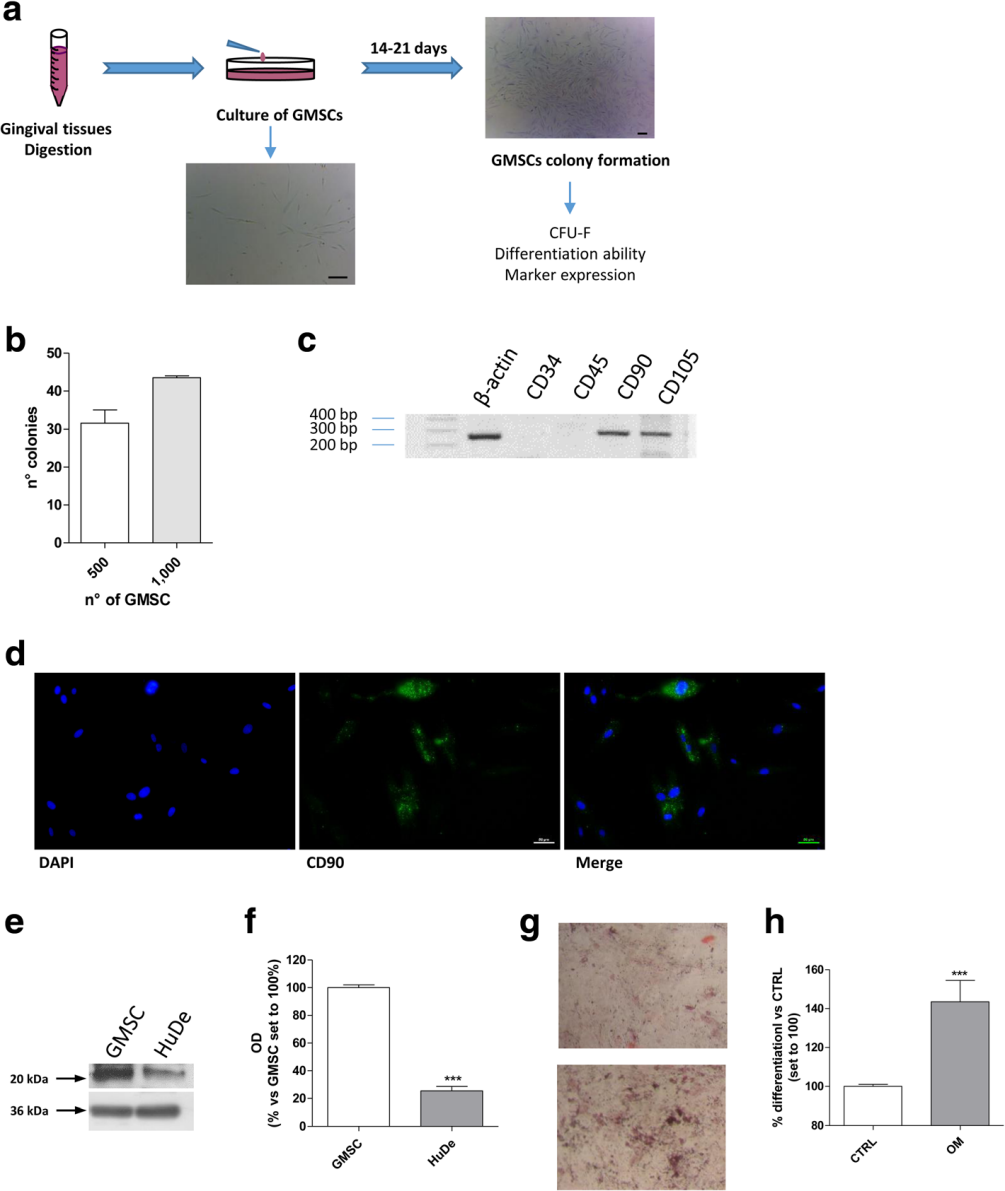
GMSC isolation and characterization. **a** Gingival tissues were digested and cultured for 14–21 days to obtain a selected population of GMSCs. **b** Colony-forming unit fibroblast (CFU-F) assay. Cells were plated at densities of 500 or 1000 cells/well. After 14 days, the cells were fixed, and stained with crystal violet. Groups of 50 or more cells were scored as colonies. The data are expressed as number of colonies, and are presented as the mean values ± SEM of three independent experiments, each performed in duplicate. **c** GMSC mRNA was extracted and PCR analysis of CD34, CD45, CD90, and CD105 was performed. Representative image of 2% agarose gel electrophoresis is shown. **d** GMSCs were fixed to determine the CD90 expression by immunofluorescence staining with anti-CD90 antibody. DAPI was used to label cell nuclei. Representative images are shown. **e**, **f** Expression of CD90 in GMSC and HuDe cells were evaluated by Western blot and a representative image (**e**) and quantitative analysis performed using ImageJ (**f**) are reported. The data were expressed as the percentage of optical density (OD) versus control set to 100% and represent the mean values ± SEM of three different experiments. The significance of the differences was determined by student *t* test: ***P ≤ 0.001 versus GMSCs. **g**, **h** The osteogenic differentiation potential was evaluated using Alizarin Red staining. GMSCs were cultured in osteogenic differentiation media for 21 days; at the end, the cells were stained and a representative photograph (10× magnified images) of mineral nodules was captured. Top represents the mineralization when GMSC were mantained in growth medium, bottom after 21 days of differentiation. (**g**). The amount of Alizarin Red S was quantified. The data are expressed as the percentage of osteogenic differentiation versus cells cultured in proliferation medium, and are presented as the mean values ± SEM of three independent experiments, each performed in duplicate. The significance of the differences was determined by student *t* test: ***P ≤ 0.001 vs. the control (CTRL)

GMSCs were isolated from gingival tissues from four healthy female subjects (average age 35.5 years). The cells were maintained in culture for 14–21 days and characterized for their phenotypic and genotypic profile. The primary cultures of single-cell suspensions exhibited a fibroblast-like spindle cell shape and formed MSC-like colonies after 10–14 days of culture at a low density (Fig. 1a). The GMSCs were analysed for their ability to form CFU-Fs, and they produced a concentration-dependent increase in colony formation of seeded GMSCs (Fig. 1b), confirming that the gingival tissue-derived cells were clonogenic.

The isolated GMSCs exhibited long-term proliferation capacity exceeding 12 passages in culture, and the doubling-time at passage 4 was 35.7 ± 3.7 h in accordance with literature data [33, 36].

The presence of specific GMSC markers were evaluated by PCR analysis (Fig. 1c); the specific MSC-associated surface markers CD90 and CD105 were highly expressed. Conversely, the hematopoietic stem cell markers CD34 and CD45 were not expressed (Fig. 1c). The expression of the specific GMSC marker CD90 was verified by immunofluorescence (Fig. 1d) and Western blot analysis (Fig. 1e, f). The CD90 protein expression was significantly higher in isolated GMSCs with respect to the fibroblast cell line (HuDe) (25.4 ± 3.2% vs. GMSC, *P* ≤ 0.001; Fig. 1e, f). These results demonstrated that the isolated cells shared a similar expression pattern with that of MSCs derived from other tissues, such as from bone marrow and dental pulp [36, 37].

GMSCs are characterized by their ability to differentiate; thus, the osteogenic potential of the isolated cells was examined. In the presence of osteogenic factors, the gingival cells formed mineralized nodules or aggregates (Fig. 1g, h). Calcium mineralization was confirmed by Alizarin Red S staining that showed a significant increase in osteogenic differentiation with respect to cells cultured in growth medium (143.5% ± 11.0 vs. control, *P* ≤ 0.01; Fig. 1h). Since these cells showed all the characteristics of human MSCs, including phenotype, self-renewal ability, and differentiation potential, these cells were designated as the GMSC population.

### TNF-α effects on GMSCs and HMEC-1 cells

**Fig. 2:**
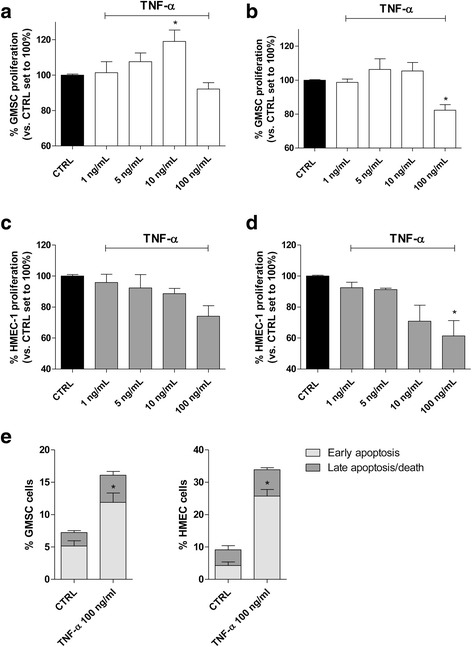
TNF-α effects on GMSC and HMEC-1 cell proliferation. GMSCs were treated in growth medium with different concentrations of tumour necrosis factor (TNF)-α (1 ng/mL to 100 ng/mL) for **a** 48 h or **b** 72 h. Human microvascular endothelial cell (HMEC)-1 cells were treated in growth medium with different concentrations of TNF-α (1 ng/mL to 100 ng/mL) for (**c**) 48 h or (**d**) 72 h. At the end of the treatments, the cell proliferation was evaluated using the MTS assay, as described in the Methods. The data are expressed as the percentage versus the untreated cells (CTRL), which was set to 100%, and are presented as the mean values ± SEM of three independent experiments, each performed in duplicate. **e** GMSCs and HMEC-1 cells were treated in the absence or presence of TNF-α (100 ng/mL) after 72 h of cell treatments. At the end, cells were collected, and the amount of phosphatidylserine externalization was evaluated using the Annexin V staining protocol. The distribution of the early and late apoptotic cells is shown. The significance of the differences was determined by one-way ANOVA, followed by Bonferroni’s post-hoc test: *P ≤ 0.05 vs. control

The effects of a wide range of TNF-α concentrations (0.1 ng/L to 100 ng/mL) on GMSC proliferation and viability were evaluated after 48 or 72 h of cell treatment (Fig. 2a, b and Additional file 1: Figure S1). The results showed that TNF-α was able to slightly increase the GMSC proliferation at a low concentration (5–10 ng/ml) after 48 h. This effect was lost after 72 h of treatment. Conversely, a high TNF-α concentration (100 ng/ml) slightly decreased the GMSC proliferation after 48 h and become significant after 72 h of treatment (*P* ≤ 0.05). Moreover, a neutral red assay demonstrated that the cytokine significantly affects the numbers of living cells only after 72 h of cell treatment, with a higher effect when the 100 ng/mL concentration was used (Additional file 1: Figure S1). These data revealed that, under our experimental conditions, TNF-α presented paradoxical effects on GMSCs dependent on cell exposure time and cytokine concentration.

Neovascularization is necessary during all the phases of wound healing. This occurs through endothelial cell activation, proliferation, and migration [38, 39]. In this process, a pivotal role is played by the MSCs that orchestrate the endothelial function through the release of different cytokines and growth factors. In this respect, the effects of a wide range of TNF-α concentrations (0.1 ng/mL to 100 ng/mL) on HMEC-1 proliferation were evaluated after 48 or 72 h of cell treatment (Fig. 2c, d). The results showed that TNF-α (100 ng/mL) slightly decreased the HMEC-1 proliferation after 48 h, with this effect becoming significant after 72 h of treatment (Fig. 2d), in accord with literature data [40]. The inflammatory cytokine did not show any positive effects on endothelial cells, but a high TNF-α concentration for a long period of exposure could decrease the cell proliferation in accord with the effects exerted on GMSCs.

The mechanism underlying the cell proliferation decrease was then investigated by evaluating the TNF-α (100 ng/mL) apoptotic activity on GMSCs and HMEC-1 cells (Fig. 2e and Additional file 1: Figure S2). The 72 h of treatment with a high TNF-α concentration was able to induce a significant phosphatidylserine externalization in the absence of 7-AAD staining (*P* ≤ 0.05), thus denoting the signs of the early phase of apoptosis in both GMSCs and endothelial cells (Fig. 2e).

### Modulation of TNF-α on GMSC cytokine and growth factor release

**Fig. 3:**
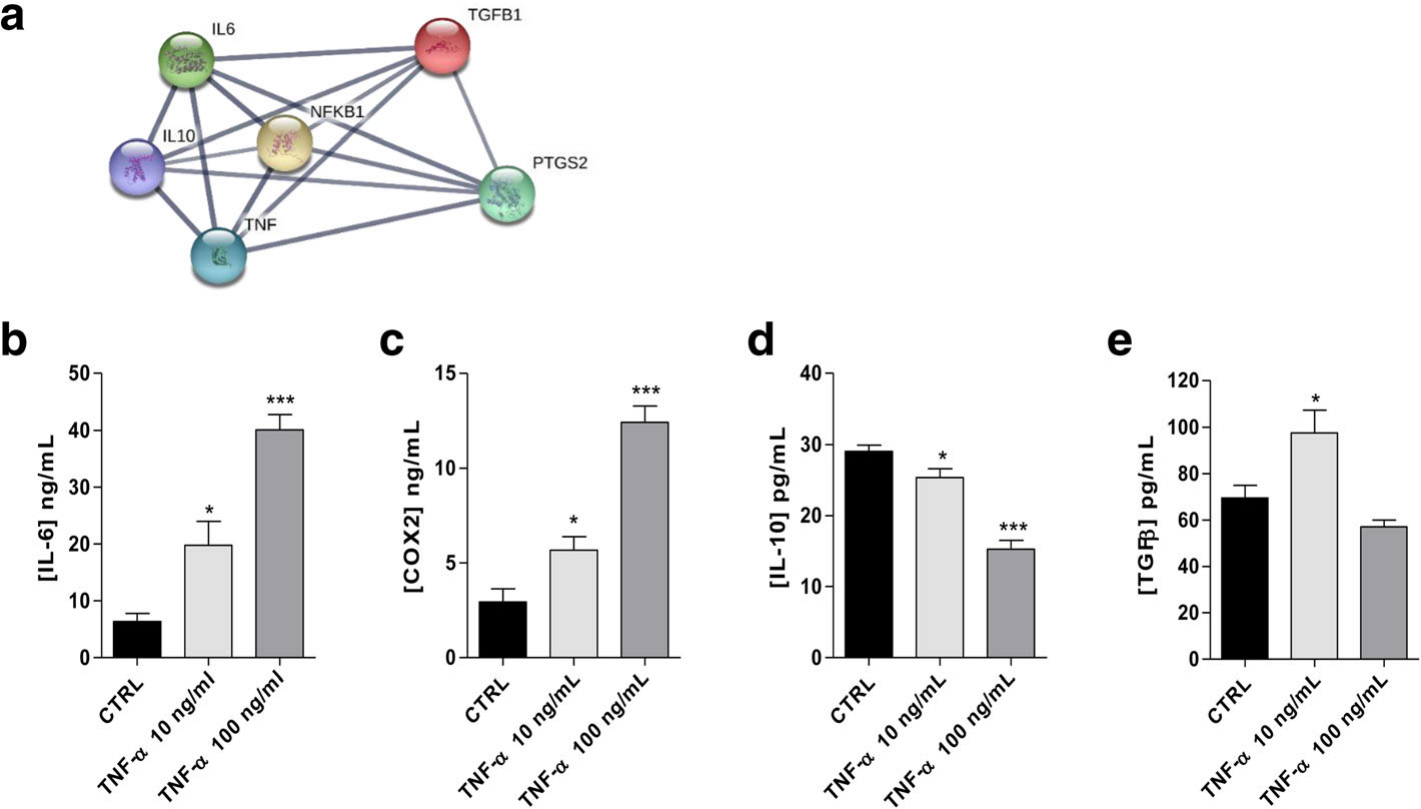
GMSC cytokine release under inflammatory conditions. **a** Interaction between NF-kB, tumour necrosis factor (TNF)-α and cytokines (interleukin (IL)-6, IL-10, PTGS2 or cyclooxygenase (COX)-2 and transforming growth factor (TGF)-β1) performed at http://string-db.org/. **b**–**e **GMSCs were treated in growth medium with different concentrations of TNF-α (10 ng/mL, 100 ng/mL) for 24 h. At the end of the treatments, the membrane COX-2 and the IL-6, IL-10, and TGF- β1 levels in the medium were quantified using commercial ELISA kits. The data are reported as the mean values ± SEM of three independent experiments each performed in duplicate. The significance of the differences was determined by one-way ANOVA, followed by Bonferroni’s post-hoc test: *P ≤ 0.05, ***P ≤ 0.001 vs. the control (CTRL)

The GMSC secretome controls and modulates the activity of several other cell types. Classic growth factors and cytokines, such as TGF-β, IL-10, IL-6, and COX2, serve as paracrine control molecules secreted or packaged into extracellular vesicles, or exosomes, by GMSCs [41, 42]. A computational STRING analysis was performed to investigate the functional interaction between NF-kB, TNF-α, and the different cytokines released by the mesenchymal cells. Based on the criteria set, a network of protein-protein interactions (PPI) that linked together the NF-kB and the different cytokines/growth factors were obtained (Fig. 3a).

Hereafter, TNF-α was used at the two concentrations we previously demonstrated to exert opposite effects on GMSC proliferation (Fig. 2). Challenging GMSCs with TNF-α at 10 or 100 ng/mL caused a concentration-dependent increase in pro-inflammatory cytokine release (IL-6 and COX2; Fig. 3b, c) and a concentration-dependent decrease of the anti-inflammatory cytokine IL-10 levels (Fig. 3d). The growth factor TGF-β, which is a well-known immunosuppressive cytokine [43], presented a hermetic concentration-response course: TNF-α at 10 ng/ml concentration increased significantly the release of the factor, whereas, the higher cytokine concentration caused a slight decrease (69.5 ± 5.5, control; 97.6 ± 9.8, TNF-α 10 ng/mL; 57.1 ± 2.9, TNF-α 100 ng/mL; Fig. 3e) of the same factor.

### TNF-α modulation of GMSC and endothelial cell interplay

**Fig. 4:**
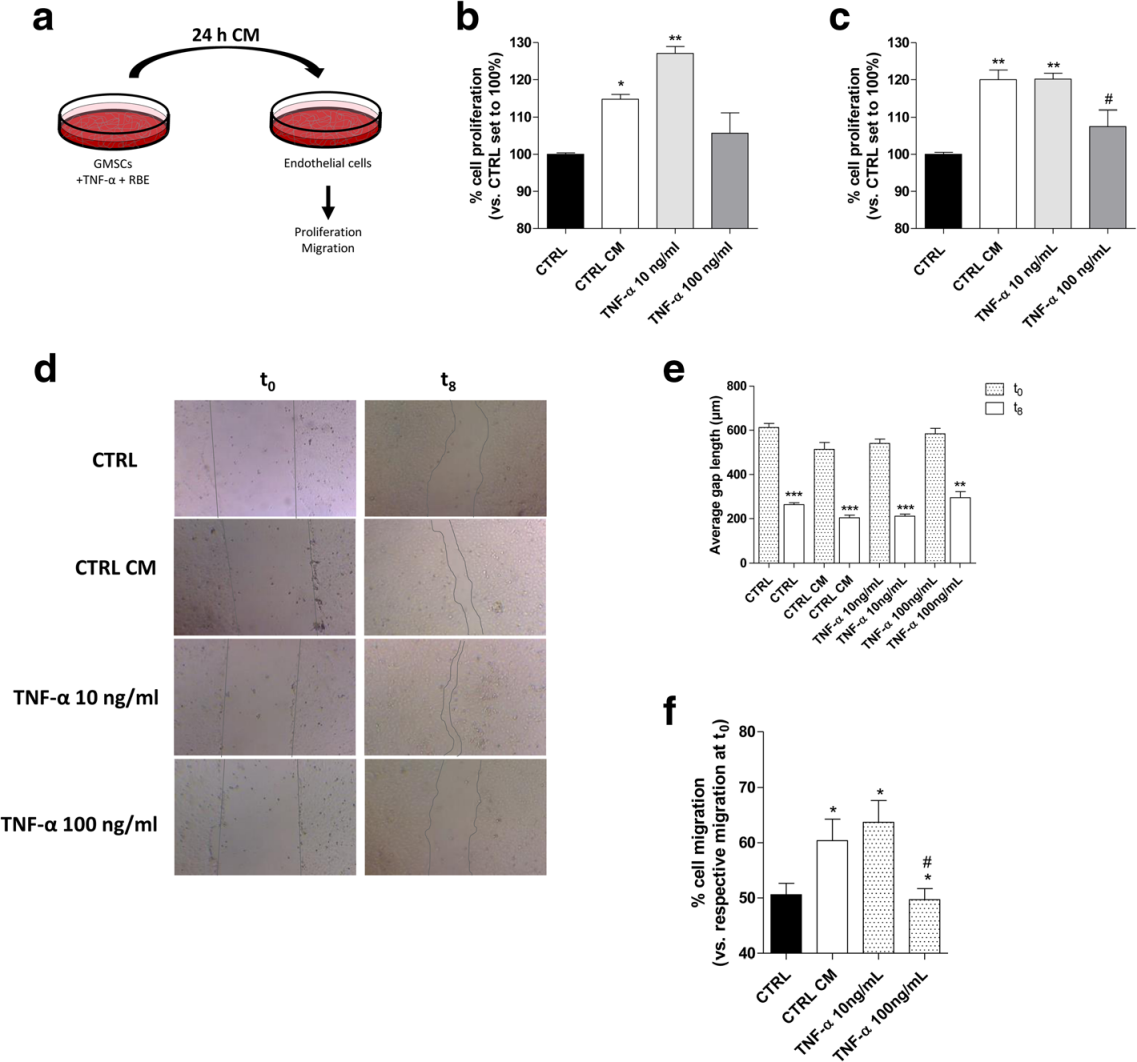
Effects of GMSC-conditioned medium (CM) on HMEC-1 proliferation and motility. **a** Schematic representation of the method. Human microvascular endothelial cell (HMEC)-1 cells were grown in 80% HMEC-1 culture medium + 20% CM obtained from control and treated GMSCs, as reported in the Methods, for **b** 24 h or **c** 48 h. At the end of the treatments, the cell proliferation was evaluated using the MTS assay. The data are expressed as the percentage versus the untreated cells (CTRL), which was set to 100%, and they are presented as the mean values ± SEM of three independent experiments, each performed in duplicate. **d**, **e** HMEC-1 cells were treated as above, and representative images of the scratch wounds at 0 h and 8 h are shown (**d**). **e** The average length of the gaps of five scratch wounds was initially measured at 0 h (t_0_) and then after 8 h (t_8_). The data are presented as the mean values ± SEM of at least two independent experiments performed in triplicate. **P ≤ 0.01, ***P ≤ 0.001 vs. the respective average gaps at t_0_. **f** Percentage of gap closure compared with untreated cells (CTRL). The data are presented as the mean values ± SEM of at least two independent experiments performed in triplicate. The significance of the differences was determined by one-way ANOVA, followed by Bonferroni’s post-hoc test: *P ≤ 0.05 vs. control; ^#^*P* ≤ 0.05 vs. the control CM (CTRL-CM). RBE, *Ribes nigrum* bud extract; TNF, tumour necrosis factor

The GMSC secretome inhibits inflammatory responses, promotes endothelial and fibroblast activities, and facilitates the proliferation and differentiation of different cells [44]. To investigate the effects of the GMSC secretome on the endothelial cell functions, HMEC-1 were treated with the conditioned medium (CM) derived by the GMSCs (Fig. 4a). In particular, GMSCs were treated for 24 h with a low (10 ng/ml) and high (100 ng/ml) concentration of TNF-α; the collected medium was applied to HMEC-1 and, then, the endothelial cell proliferation and motility were analysed.

The CM derived by the GMSCs without treatment (control CM) was able to promote endothelial cell proliferation after 24 h (Fig. 4b) and, more efficiently, after 48 h of cell treatment (120.6 ± 2.6% vs control; Fig. 4b). Furthermore, control CM was able to significantly increase the endothelial cell motility (Fig. 4d–f). These data demonstrate that the GMSCs, per se, were able to exert a trophic activity on the endothelial cells in accordance with the reported trophic properties of GMSCs [18].

Next, the effects of TNF-α on the GMSC-HMEC-1 interplay were evaluated. A high concentration of TNF-α (100 ng/mL) was able to modify the GMSC secretome, producing a significant negative effect on HMEC-1 proliferation (120.6 ± 2.6%, CM; 107.5 ± 4.4%, TNF-α; Fig. 4b, c) and motility (56.4 ± 0.8%, CM; 49.7 ± 2.0%, TNF-α; Fig. 4e, f). Conversely, no significant effects on HMEC-1 well-being or functionality were observed with a low dose of the cytokine, despite a positive trend in the promotion of endothelial motility being evident (Fig. 4). These results demonstrate that cytokines such as TNF-α could turn off the beneficial and trophic effects of GMSCs, negatively affecting their interaction with surrounding cells dependent on time and concentration.

### Chemical composition of the bud preparation

**Fig. 5:**
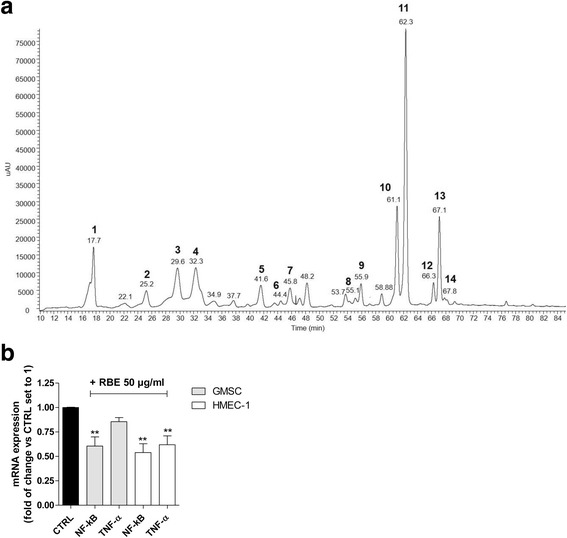
Composition and anti-inflammatory effect of RBE. **a** LC-PDA/UV chromatogram (detected at 325 nm) of *Ribes nigrum* bud extract (RBE) preparation. For peak data, see Table 2. **b** GMSCs or human microvascular endothelial cell (HMEC)-1 cells were treated with RBE (50 μg/mL) for 24 h. At the end of the incubation, NF-kB and tumour necrosis factor (TNF)-α expression levels were detected by real time RT-PCR. The data are expressed as the fold of change versus the control (CTRL), which were set to 1, and are presented as the mean values ± SEM of three independent experiments. The significance of the differences was determined by one-way ANOVA, followed by Bonferroni’s post-hoc test: **P ≤ 0.01, vs. control

The chemical composition of the RBE hydro-alcoholic glycerine solution was investigated by means of HPLC-PDA/UV-ESI-MS/MS techniques. The LC-PDA/UV chromatogram, acquired at 325 nm, is shown in Fig. 5. Compounds 1–14 (in Fig. 5a) were identified by comparison with their elution orders, ESI-MS/MS data, and PDA/UV absorbance (Table 2) with data reported in the literature. All identified molecules belong to the phenols class, including phenolic acid derivatives, such as chlorogenic acids (1, 3, and 4 in Fig. 5a), *p*-coumaroylquinic acids (2, 5, and 7 in Fig. 5a), and flavonol glycosides, such as myricetin 3-*O*-rutinoside (8 in Fig. 5a), myricetin 3-*O*-glucoside and/or myricetin 3-*O*-galactoside (9 in Fig. 5a), quercetin 3-*O*-rutinoside (10 in Fig. 5a), quercetin 3-*O*-glucoside and/or quercetin 3-*O*-galactoside (11 in Fig. 5a), kaempferol 3-*O*-rutinoside (12 in Fig. 5a), kaempferol 3-*O*-glucoside (13 in Fig. 5a), and isorhamnetin glucoside and/or isorhamnetin galactoside (14 in Fig. 5a).

**Table 2:** Spectral (UV and ESI-MS/MS) and chromatographic data (retention time, *t*_R_) of phenols (1–14) detected in *R. nigrum* bud preparation

Peak	Compound	*t*_R_ (min)	M	[M − H]^−^	MS/MS base peak (*m/z*)	MS/MS ions (*m/z*)	λ_max_ (nm)
	*Phenolic acids*						
1	Caffeoylquinic acid	17.7	354	353	179	191, 173, 135	257, 325
2	3-*p*-Coumaroylquinic acid	25.2	338	337	163	293, 203, 191,173	313
3	Caffeoylquinic acid	29.6	354	353	179	191, 173, 155, 135	257, 329
4	Caffeoylquinic acid	32.3	354	353	191	179, 173, 135	257, 329
5	4-*p*-Coumaroylquinic acid	41.6	338	337	173	293, 191, 173, 163, 155	313
7	5-*p*-Coumaroylquinic acid	45.8	338	337	191	293, 207, 173, 163	314
	*Flavonol glycosides*						
6	Kaempferol di-hexoside	44.4	610	609	447	573, 489, 285, 179	270, 356
8	Myricetin 3-*O*-rutinoside	55.1	626	625	317	607, 479, 271, 243, 203, 179	270, 357
9	Myricetin 3-*O*-glucoside and/or myricetin 3-*O*-galactoside	55.9	480	479	317	461, 271, 179, 151	269, 358
10	Quercetin 3-*O*-rutinoside (rutin)	61.1	610	609	301	591, 447, 271, 243, 179	265, 356
11	Quercetin 3-*O*-glucoside (isoquercitrin) and/or quercetin 3-*O*-galactoside (hyperoside)	62.3	464	463	301	445, 273, 179	257, 356
12	Kaempferol 3-*O*-rutinoside	66.3	594	593	285	447, 257, 229, 179	270, 350
13	Kaempferol 3-*O*-glucoside (astragalin)	67.1	448	447	285	257, 229, 179	268, 349
14	Isorhamnetin glucoside and/or isorhamnetin galactoside	67.8	478	477	315	285, 271, 243, 229, 179	271, 356

Compound numbers correspond with peak numbers in Fig. 5

Compounds 1, 3, and 4 in Fig. 5a are three isomers showing a similar mass spectra consisting of the same parent ion [M − H]^−^ at *m/z* 353 and different fragment ions at *m/z* 191, due to the loss of the caffeoyl moiety, 179, generated by the loss of the quinic unit, 173, corresponding to a product ion [quinic acid−H − H_2_O]^−^, and 135, due to a [caffeic acid−H − CO_2_]^−^ ion. These data are in agreement with those reported for these compounds by Clifford et al. [45]. The presence of 3-*O*-caffeoylquinic acid (neochlorogenic acid), 4-*O*-caffeoylquinic acid (cryptochlorogenic acid), and 5-*O*-caffeoylquinic acid (chlorogenic acid) in RBE buds was recently reported [46, 47]. Peaks 2, 5, and 7 in Fig. 5a (λ_max_ 313–314) displayed the same full mass spectra with deprotonated molecules [M − H]^−^ at *m/z* 337. MS/MS spectra have a similar fragmentation pathway, showing fragment ions at *m/z* 173, due to the loss of a *p*-coumaric acid molecule ([M − 164]^−^), and two other diagnostic ions at *m/z* 191 and 163, in accord with the fragmentation profile of *p*-coumaroylquinic acids [48]. These finding are in agreement with the study of Ieri et al. who reported the presence of *p*-coumaroylquinic acid isomers in commercial bud preparations of blackcurrant [47]. It is possible to discriminate between each of the three isomers on the basis of the base peak value generated in the MS/MS spectra, according to Clifford et al. [48]. Indeed, base peaks at *m/z* 163, 173, and 191 were generated by fragmentation of *p*-coumaroylquinic acid parent ions carrying the esterification at position 3, 4, and 5, respectively. Thus, compounds 2, 5, and 7 could be identified as 3-*p*-coumaroylquinic acid, 4-*p*-coumaroylquinic acid, and 5-*p*-coumaroylquinic acid, respectively.

Peaks 8–14 in Fig. 5a were assigned to flavonoid glycosides showing characteristic UV spectra, with two strong absorption peaks at 255–270 and 349–358 nm, typical of a flavonol structure. Aglycon portions were represented by myricetin, kaempferol, quercetin, and isorhamnetin as deduced by fragment ions in the MS/MS spectra at *m/z* 317, 285, 301, and 315, respectively. Parent ions ([M − H]^−^) of flavonoid monoglycosides at *m/z* 479 (9 in Fig. 5a), 463 (11 in Fig. 5a), 447 (13 in Fig. 5a), and 477 (14 in Fig. 5a) displayed product ions generated by the loss of one hexose moiety ([M − 162]^−^) due to the cleavage of *O*-sugar bond. MS/MS spectra of compounds 8 and 10 in Fig. 5a showed product ions generated by the loss of one rhamnose moiety ([M − 146]^−^) and one hexose moiety ([M − 162]^−^) attributable to the presence of the disaccharide rutinose linked to the myricetin and quercetin aglycons, respectively. Thus, compounds 8 and 10 were identified as myricetin 3-*O*-rutinoside and quercetin 3-*O*-rutinoside, respectively. The detection of all these bioactive constituents was in agreement with results previously reported [46, 47].

The MS/MS experiment for peak 6 in Fig. 5a ([M − H]^−^ at *m/z* 609) provided product ions at *m/z* 447 and 285, generated by the subsequent losses of two hexose moieties, not identifiable through spectral data; thus compound 6 was supposed to be a kaempferol di-hexoside by PDA/UV and MS/MS data [49].

### *R. nigrum* anti-inflammatory effects on GMSCs and HMEC cells

The anti-inflammatory properties of RBE [31], and the effects of some of its constituents on inflammatory gene transcription such as NF-kB and TNF-α [50–52], have been demonstrated. Inflammation is a pivotal process that may positively or negatively affect regenerative processes and the different cell types involved [53]. Thus, the ability of the RBE to modulate NF-kB and TNF-α gene transcription was evaluated in both GMSCs and HMECs (Fig. 5b).

Challenging the GMSCs with the RBE (50 μg/mL) for 24 h produced a significant decrease in NF-kB (0.60 ± 0.09 fold with respect to control, *P* ≤ 0.001) gene expression, and only a slight decrease in TNF-α transcription. In HMEC-1 cells treated with RBE (50 μg/mL) for 48 h, the NF-kB expression was significantly decreased (0.54 ± 0.09 fold with respect to control, *P* ≤ 0.001) as well as the TNF-α expression (0.62 ± 0.10 fold with respect to control, *P* ≤ 0.001), which is one of the pro-inflammatory cytokines mainly affecting the endothelial cell fate [54]. Taken together, these results demonstrated that RBE has anti-inflammatory effects on GMSCs and HMEC-1 cells, negatively modulating pro-inflammatory pathways.

### Effects of *R. nigrum* on GMSC and HMEC-1 cell proliferation and well-being

**Fig. 6:**
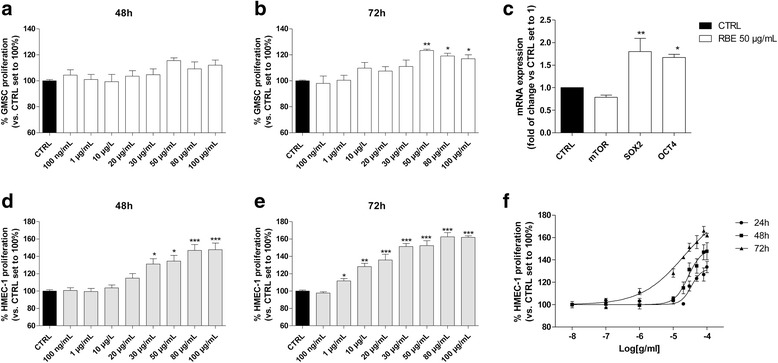
RBE effects on GMSC and HMEC-1 cell proliferation and stemness gene expression. GMSCs were treated in growth medium with different concentrations of *Ribes nigrum* bud extract (RBE; 100 ng/mL to 100 μg/mL) for **a** 48 h or **b** 72 h. At the end of the treatments, the cell proliferation was evaluated using the MTS assay. The data are expressed as the percentage versus the untreated cells (CTRL), which was set to 100%, and are presented as the mean values ± SEM of three independent experiments, each performed in duplicate. **c** GMSCs were treated with RBE (50 μg/mL) for 24 h. At the end of the incubation, a real time RT-PCR analysis of mTOR, Oct4, and SOX2 was performed. The data are expressed as the fold change versus the control (CTRL) levels (without RBE), which were set to 1, and are presented as the mean values ± SEM of three different experiments. Human microvascular endothelial cell (HMEC)-1 cells were treated in growth medium with different concentrations of RBE (100 ng/mL to 100 μg/mL) for **d** 48 h or **e** 72 h. At the end of the treatments, the cell proliferation was evaluated using the MTS assay, as described in the Methods. The data are expressed as the percentage versus the untreated cells (CTRL), which was set to 100%, and they were presented as the mean values ± SEM of three independent experiments, each performed in duplicate. **f** Non-linear regression with variable slope of the RBE concentration-response curve at the different times. The significance of the differences was determined by one-way ANOVA, followed by Bonferroni’s post-hoc test: *P ≤ 0.05, **P ≤ 0.01, ***P ≤ 0.001 vs. control

To investigate the putative effects of RBE on stem cell proliferation/viability, isolated GMSCs were treated with a wide range of the extract concentrations (100 ng/mL to 100 μg/mL) for 48 and 72 h. The results (Fig. 6a, b) showed that higher concentrations of RBE slightly affected the GMSC proliferation after 48 h of cell treatment and the effects become significant only after 72 h. The proliferative effects were dose-dependent, with a maximum increase of 123.3 ± 2.5% (50 μg/mL) of cell growth. Moreover, a neutral red assay demonstrated that the extract significantly affected the numbers of living cells only after 72 h of cell treatment, with the higher effect when the 50 μg/mL concentration was used (Additional file 1: Figure S1). These data show the ability of RBE to affect the proliferation of the GMSCs, increasing the cell number in a dose- and time-dependent manner. The induction of MSC proliferation and the maintenance of stemness features represents a pivotal mechanism for improving the regenerative properties of the MSCs [55].

The role of the transcription factors that regulate self-renewal and differentiation is well known in embryonic stem cells [56]. Among these, Oct4 (octamer-binding transcription factor 4) and SOX2 (sex determining region Y (SRY)-box 2) work together to support each other’s expression and that of other self-renewal genes repressing differentiation genes [57, 58]; thus, the effects of RBE on the expression of Oct4 and SOX2 stemness genes were investigated (Fig. 6c). In parallel, the expression of mammalian target of rapamycin (mTOR) that was involved in MSC differentiation was quantified.

GMSC treatment with RBE (50 μg/mL) did not significantly affect mTOR expression (Fig. 6c). Conversely, it was able to significantly increase the gene expression of the stemness markers Oct4 and SOX2 (1.8 ± 0.3 and 1.7 ± 0.1 fold change, respectively). Taken together, these results demonstrated the positive effect of the RBE on GMSC well-being, promoting stemness maintenance.

To investigate the effects of RBE alone on the proliferation/viability of endothelial cells (HMEC-1), cells were treated with different concentrations of RBE (100 ng/mL to 100 μg/mL) for 48 or 72 h. The results (Fig. 6d–f) showed that it affected the HMEC-1 proliferation at higher concentrations after 24 h of cell treatment in a concentration-dependent manner, with an EC_50_ value of 34.5 μg/mL. This effect become more evident after 48 and 72 h, with an increase in both the EC_50_ values (27.4 and 21.0 μg/mL, respectively) and the maximum effects (147.7 ± 7.6% and 162.0 ± 1.7%, respectively). These data show the ability of RBE to affect the proliferation of HMEC-1 cells. Interestingly, the endothelial cells showed a higher sensitivity to the extract exposure per se with respect to the GMSCs (Fig. 6a, b).

### *R. nigrum* decrease the negative effects of TNF-α in GMSCs and HMEC-1 cells

**Fig. 7:**
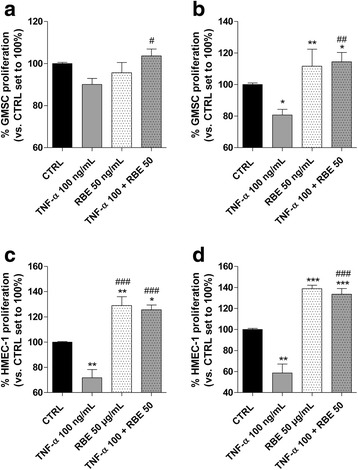
RBE modulation of TNF-α activity on GMSCs and HMEC-1 cells. GMSCs were treated in growth medium with different concentrations of tumour necrosis factor (TNF)-α (1 ng/mL to 100 ng/mL) in the absence or the presence of the *Ribes nigrum* bud extract (RBE; 50 μg/mL) for **a** 48 h or **b** 72 h. Human microvascular endothelial cell (HMEC)-1 cells were treated in growth medium with different concentrations of TNF-α (1 ng/mL to 100 ng/mL) in the absence or the presence of RBE (50 μg/mL) for **c** 48 h or **d** 72 h. At the end of the treatments, the cell proliferation was evaluated using the MTS assay, as described in the Methods. The data are expressed as the percentage with respect to the untreated cells (CTRL), which was set to 100%, and are presented as the mean values ± SEM of three independent experiments, each performed in duplicate. The significance of the differences was determined by one-way ANOVA, followed by Bonferroni’s post-hoc test or student *t* test: *P ≤ 0.05, **P ≤ 0.01 vs. control (CTRL); ^#^*P* ≤ 0.05, ^##^*P* ≤ 0.01 vs. the respective TNF-α

To fully investigate the effects of TNF-α on GMSC trophic activity, the RBE was used as anti-inflammatory agent since it had been demonstrated to interfere with the inflammatory pathways in GMSCs and HMEC-1 cells, promoting their well-being. RBE cell treatment for 72 h in the presence of 100 ng/ml TNF-α completely counteracted the cytokine effects on GMSC proliferation, restoring the cell growth rate as demonstrated by MTS and neutral red assays (Fig. 7a, b and Additional file 1: Figure S2).

Next, the effects of RBE on TNF-α-induced blockade of HMEC-1 cell proliferation and viability were evaluated (Fig. 7c, d). The extract per se increased the HMEC-1 viability; interestingly, RBE was able to completely counteract the negative effects of 100 ng/mL TNF-α, restoring the cell growth rate. Taken together, these results suggest that RBE exerts a cyto-protective effect in an experimental model of inflammation counteracting the negative effects exerted by a high TNF-α concentration, both in human GMSCs and, more markedly, in endothelial cells.

### Restoration of GMSC cytokine and growth factor release by *R. nigrum*

**Fig. 8:**
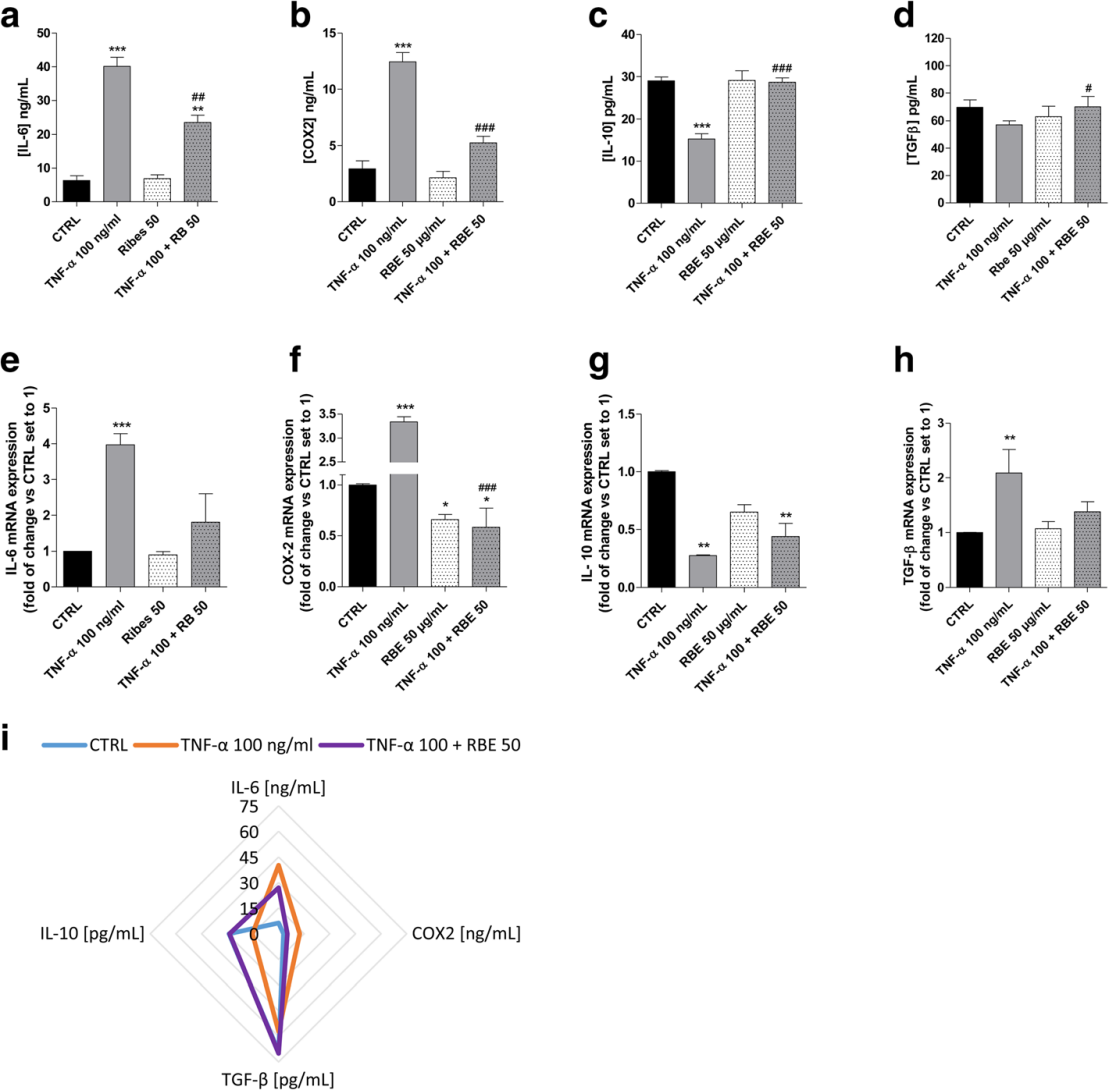
RBE modulation of GMSC cytokine release under normal and inflammatory conditions. **a**–**h** GMSCs were treated in growth medium with different concentrations of tumour necrosis factor (TNF)-α (10 ng/mL, 100 ng/mL) in the absence or the presence of *Ribes nigrum* bud extract (RBE; 50 μg/mL) for 24 h. **a**–**d** At the end of the treatments, the membrane cyclooxygenase (COX)-2 and the interleukin (IL)-6, IL-10, and transforming growth factor (TGF)-β1 levels in the medium were quantified using ELISA kits. The values are presented as the mean values ± SEM of three different experiments. **e**–**h** GMSCs, treated as above, were lysed and real-time RT-PCR analysis of IL-6, IL-10, COX-2, and TGF- β1 was performed. The data are expressed as the fold of change versus the untreated cells (CTRL), which were set to 1, and are presented the mean values ± SEM of three different experiments. The significance of the differences was determined by one-way ANOVA, followed by Bonferroni’s post-hoc test: *P ≤ 0.05, **P ≤ 0.01, ***P ≤ 0.001 vs. control (CTRL); ^#^*P* ≤ 0.05, ^##^*P* ≤ 0.01, ^###^*P* ≤ 0.001 vs. the respective TNF-α. **i** The radar plot shift of the cytokine production

First, the putative effects of RBE alone on the GMSC secretome were investigated (Fig. 8). Isolated GMSCs were treated with RBE (50 μg/mL) for 24 h and quantitative analysis of the released cytokines was performed. The unstimulated GMSCs secreted low levels of anti-inflammatory molecules (TGF-β and IL-10), and RBE alone was not able to alter their expression pattern (Fig. 8). Thus, RBE per se was not able to alter the trophic function of GMSCs.

Then, GMSCs were treated with an inflammatory stimulus (TNF-α 100 ng/mL) in the presence of RBE. The presence of RBE was able to significantly reduce the release of IL-6 and the expression of COX2, with levels comparable with that obtained with a low TNF-α (10 ng/mL) stimulation. RBE significantly increased IL-10 release and expression. Finally, RBE was able to only partially increase TGF-β. Furthermore, in a radar plot, a shift of the cytokine production versus the basal condition was shown (Fig. 8i). These results highlight that RBE is able to modulate the inflammatory process. Furthermore, the extract did not completely block the cytokine release but only restored the levels necessary to mediate the positive effect of a controlled inflammation.

### GMSC and endothelial cell interplay is modulated by *R. nigrum* extract

**Fig. 9:**
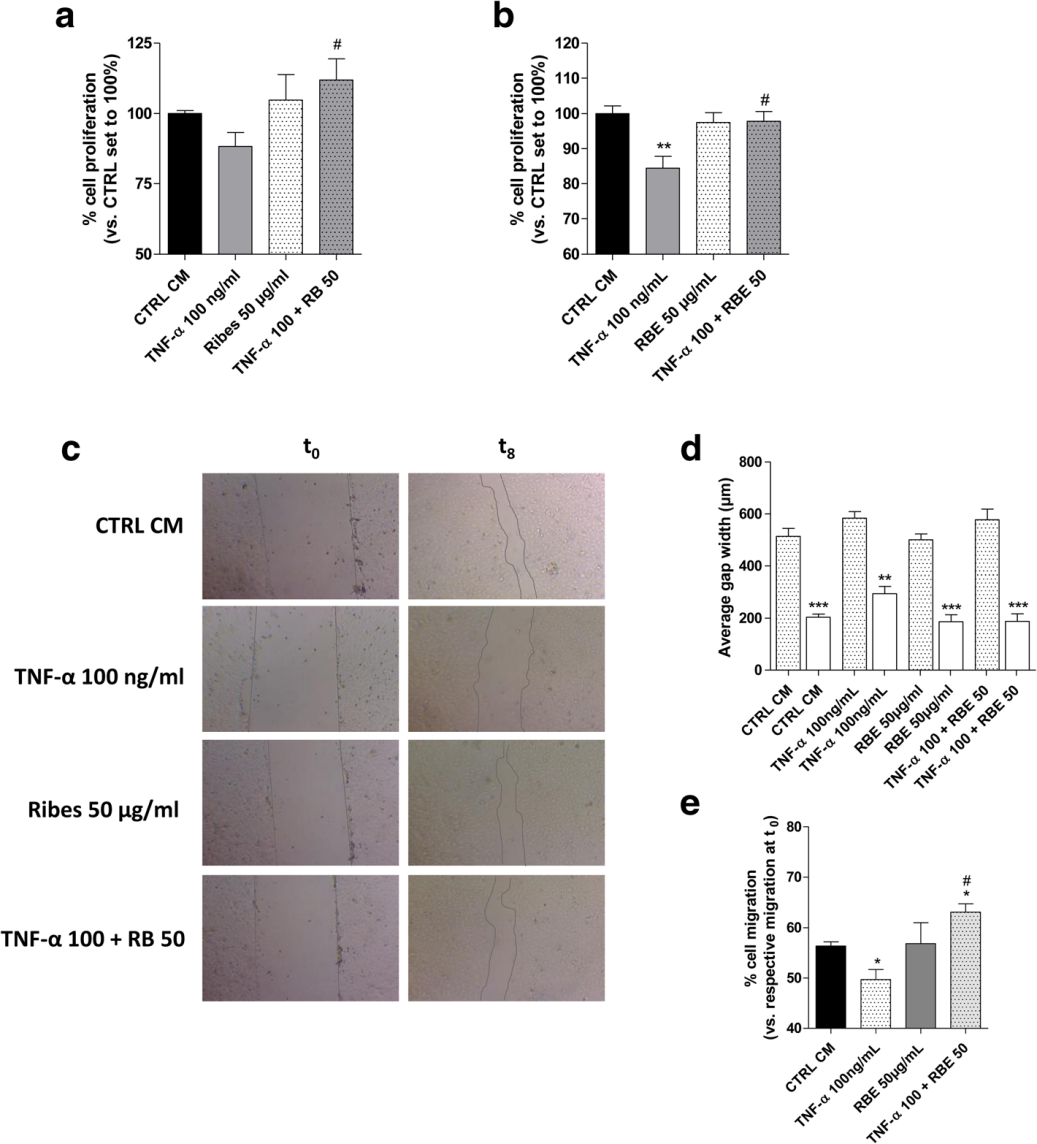
Modulatory effects of *Ribes nigrum* bud extract (RBE) on GMSC-HMEC-1 interplay. **a**, **b** HMEC-1 cells were grown in 80% HMEC-1 culture medium + 20% conditioned medium (CM) obtained from control (CTRL) and treated GMSCs, as reported in the Methods, for **a** 24 h or **b** 48 h. At the end of the treatments, the cell proliferation was evaluated using the MTS assay. The data are expressed as the percentage versus the control cells (CTRL CM), which was set to 100%, and are presented as the mean values ± SEM of three independent experiments, each performed in duplicate. **c** HMEC-1 cells were treated as above, and representative images of the scratch wounds at 0 h and 8 h are shown. **d** The average length of the gaps of five scratch wounds was initially measured at 0 h (t_0_) and then after 8 h (t_8_). The data are presented as the mean values ± SEM of at least two different experiments performed in triplicate. **P ≤ 0.01, ***P ≤ 0.001 vs. the respective average gaps at t_0_. **e** Percentage of gap closure compared to control cells (CTRL). The data are presented as the mean values ± SEM of at least two different experiments performed in triplicate. The significance of the differences was determined by one-way ANOVA, followed by Bonferroni’s post-hoc test: *P ≤ 0.05, **P ≤ 0.01 vs. control; ^#^*P* ≤ 0.05 vs. tumour necrosis factor (TNF)-α 100 ng/mL

Considering that RBE promotes anti-inflammatory actions through the modulation of the GMSC secretome, the extract effects on GMSC-HMEC-1 cell interplay was evaluated by the CM method (Fig. 9). The extract alone did not modify the HMEC-1 cell proliferation (Fig. 9a, b) in accord with the absence of the GMSC secretome composition; interestingly, RBE was able to completely counteract the negative effects of TNF-α (100 ng/mL) on HMEC-1 cells, restoring the levels of cell growth rate triggered by GMSC control CM. GMSC-CM and TNF-α (100 ng/mL) produced comparable effects on HMEC-1 motility to that reported above (Fig. 4d–f). Furthermore, the treatment of GMSCs with the extract alone produced a CM that did not modify endothelial cell motility (Fig. 9c–e), in agreement with the lack of effects on GMSC cytokine release (Fig. 8). Surprisingly, in the presence of a highly inflammatory microenvironment, RBE was able to significantly counteract the negative effects of the high concentration of TNF-α. These results suggest that the use of natural agents, such as RBE, are able to counteract the effects of a high TNF-α concentration, restoring the GMSC secretome similarly to that obtained in controlled inflammation (a low TNF-α dose) which could effectively be used to modulate the GMSC response during the regenerative process. In particular, these results demonstrate a necessity for controlling the inflammatory priming of GMSCs during the regenerative process both in vivo and in vitro when GMSCs are used in engineering grafts.

## Discussion

Recently, great attention has been paid to the use of MSCs in regenerative medicine and to the discovery of agents able to modify the well-being and functionality of these particular kinds of cells [18]. In this study, the effects of TNF-α used at different concentrations were evaluated on GMSC and endothelial cell well-being, demonstrating its paradoxical effects. Low concentrations positively enhanced the well-being of these cells that are pivotal actors in tissue repair; conversely, a high concentration promoted the release of high amounts of inflammatory cytokines thus negatively affecting the regenerative properties of GMSCs. Furthermore, we demonstrated that the use of natural compounds, such as RBE which were able to modulate the inflammatory pathways, counteracted the negative influence of TNF-α on GMSC-endothelial cell cross-talk, modulating the GMSC secretome composition probably through the modulation of NF-kB gene expression.

The role of MSCs in the regenerative process has been widely studied [59]. Despite the main function of MSCs is their differentiation ability, the therapeutic effects of MSCs may also depend on their capability to regulate inflammation and tissue homeostasis [16, 55, 60]. Presently, BM is considered as a prime source of MSCs, although their isolation is an invasive procedure for both patients and donors. BM-MSCs enter senescence and lose their stem cell characteristics early in culture [61]. Thus, several studies have been undertaken to find appropriate alternatives. Gingiva is a tissue with a high regenerative capacity and has been highlighted as one possible source of MSCs [18, 62]. Considering the function and characteristics of GMSCs, they have become an exciting alternative for tissue engineering approaches, aiming to enhance wound repair in oral and extra-oral tissues [18]. In fact, the GMSCs are able to accelerate the repair process in a murine excisional full-thickness skin wound model [63]. Herein, the GMSC cultures were easily obtained by biopsies from healthy subjects, and the isolation procedure lead to a homogenous culture of mesenchymal cells as previously described [33, 36]. These cells could differentiate in bone and express the typical surface markers of mesenchymal cells, in accordance with the literature data [61].

Inflammation is a pivotal phase in normal tissue repair; however, pathologically chronic and extensive inflammation causes detrimental effects leading to the disruption of the normal healing cascade [64]. MSCs have been widely described as the guardian of the inflammatory process due to their ability to modulate the immune response and promote wound healing [65]. Accordingly, it has been demonstrated that the pro-inflammatory cytokine TNF-α exerts opposite and conflicting effects on MSCs, primarily depending on its concentration and time of cell exposure [66–68]. In our experimental model, a low TNF-α concentration (10 ng/mL) or a low time of cell exposure (24–48 h) does not negatively affect the proliferation of GMSCs, in accordance with the literature [69]. In gingival tissue, chronic inflammation is one of the main causes of gingival hyperplasia that is the result of robust cell proliferation [70]. However, the amounts of inflammatory cytokines, such as TNF-α, even if they are significantly increased in gingival tissue of gingivitis and periodontitis patients, remains in the low nanomolar/picomolar range [71]. This supports the positive effects exerted by a low TNF-α concentration on GMSC proliferation in our cellular model. Conversely, a high concentration of the cytokine (100 ng/mL) for a longer time (72 h), which could mimic a prolonged exposure to a pathological inflammatory state, produces a significant decrease in cell proliferation. It has been reported that TNF-α induces apoptosis in several cell types [72–75]. In our cellular model, the decrease in GMSC proliferation could be explained by a slight increase in apoptotic processes, despite the possibility of other mechanisms of cell death that could not be excluded. In fact, MSCs, when maintained in culture, undergo a premature senescence, thus limiting their use in cell therapy [76]. The use of external stressor such as radiation, reactive oxygen species (ROS), and cytokines could promote not only the apoptotic process but also senescence in MSCs [77, 78]. Further studies are in progress to fully investigate the possible role of TNF-α in induced-GMSC senescence.

Recently, increasing evidence indicates that MSCs exert their beneficial effects by secreting paracrine or trophic factors [55], as demonstrated by the ability of MSC-conditioned media to induce wound healing [79]. MSC-conditioned medium contains several cytokines, growth factors, and exosomes, containing miRNAs, mRNAs, and proteins, that can be transferred as a type of “physiological lipofection” to recipient cells and modifying their characteristics [80–82]. In this respect, it has been demonstrated that the trophic role of the GMSCs is mainly due to the modulation of cytokines such as IL-6, TGF-β, prostaglandin E2, and vascular endothelial growth factor (VEGF) [2, 83]. These secreted factors may inhibit inflammatory responses, promote fibroblast activities, facilitate the proliferation and differentiation of progenitor cells in tissues, and promote angiogenesis which is necessary to support the regenerative process by maintaining an adequate tissue oxygenation [44, 84]. In our experimental setting, challenging GMSCs with a low TNF-α (10 ng/mL) concentration promoted the secretion of low amounts of pro-inflammatory cytokines and increased the release of TGF-β leading to a positive effect on endothelial proliferation and motility. Conversely, a high concentration of TNF-α significantly modified the release of different cytokines and growth factors (IL-6, IL-10, TGF-β, and COX-2) towards a pro-inflammatory microenvironment. The modification of the GMSC secretome produced a negative effect on endothelial cell function (proliferation and motility). These results reflect the well-established opposite effect of TNF-α, demonstrating the importance of controlling the cytokine concentration in in-vitro applications to promote the function of MSCs [85] and to control the inflammatory microenvironment during the wound-healing process.

Different approaches have been utilized to harness the trophic function of MSCs [86]. Numerous innovative and conventional biological agents, including traditional oriental herbal medicines, have recently been tested for their preconditioning effect in vitro in an attempt to improve the cellular properties and regenerative treatment outcome of GMSCs in vivo [18]. Extracts of plants and fruits such as *Ligustrum lucidum*, *Herba epimedii*, or *Cissus quadrangularis* have been demonstrated to affect the differentiation/proliferation and trophic activity of MSCs [87]. However, to date no data have been reported on the RBE biological activity properties on GMSCs and endothelial cells.

In the current study, the chemical composition of the RBE showed a high content of phenols that have been reported to possess anti-inflammatory activity [88, 89]. Results from the HPLC-PDA/UV-ESI-MS/MS analyses revealed that RBE is rich in phenol constituents (Table 2), in agreement with data previously reported [28]. In particular, phenolic acid derivatives and flavonol mono- and di-glycosides were revealed. Compounds 1, 3, and 4 (λ_max_ 257 and 325–329 nm) were identified as caffeoylquinic acids. Compounds 2, 5, and 7 can be identified as 3-*p*-coumaroylquinic acid, 4-*p*-coumaroylquinic acid, and 5-*p*-coumaroylquinic acid, respectively. Finally, compounds 8–14 were identified as myricetin 3-*O*-rutinoside (8), myricetin 3-*O*-glucoside and/or myricetin 3-*O*-galactoside (9), quercetin 3-*O*-rutinoside or rutin (10), quercetin 3-*O*-glucoside and/or quercetin 3-*O*-galactoside (11), kaempferol 3-*O*-rutinoside (12), kaempferol 3-*O*-glucoside (13), and isorhamnetin glucoside and/or isorhamnetin galactoside (14). The detection of all these bioactive constituents was in agreement with results previously reported [46, 47].

Several flavonols have been reported to exhibit controversial effects on cell proliferation depending on the cell type and dose. Kaempferol is able to increase human fibroblast proliferation when applied at a low concentration. Conversely, at a high dose it induces a significant decrease in cell growth [90]. Similar effects have been reported for quercetin in several cell types [91–93]. Herein, for the first time, the ability of RBE to promote GMSC and endothelial cell proliferation was reported. The positive action of RBE on mesenchymal cell well-being was confirmed by the significant increase in stemness gene transcription, such as Oct4 and SOX2. In fact, it is well known that the expression of such genes is pivotal for the successful establishment of the pluripotent state and the maintenance of adult stem cells [94]. The discordance between the effects reported for the single constituents and the total extract could be explained by the difference in the constituent concentration when used alone or as part of the total extract. In fact, the concentrations of each phytochemical in the total extract remain lower, and the final biological effects could derive from the combination of the positive effects exerted by these low concentrations.

The RBE demonstrated the ability to counteract the negative effect of TNF-α, restoring the cytokine release pattern of untreated GMSCs. This is in accordance with the effects of the quercetin on orbital fibroblasts, where it is able to decrease the inflammatory-induced expression of IL-6 and COX-2, and to increase IL-10 through the decrease of NF-kB activation [95]. Similar effects have been reported for kaempferol in chondrocyte and macrophages [96, 97].

## Conclusions

Taken together, these data shed light on the role of TNF-α on GMSC well-being and trophic activity. In the scenario of regenerative medicine and in the search for pre-conditioned agents able to modify the secretome of these cells, the present study demonstrates how the extracellular microenvironment could differently affect the functionality of GMSCs dependent on time of exposure and concentration. In parallel, these data demonstrate the positive effects of RBE to enhance the well-being of the stem cells and to increase their trophic activity. Nevertheless, the effects on several other cytokines and growth factors involved in wound healing processes should be fully investigated; these data highlight the importance of modulating the inflammatory priming. Furthermore, the modulation of the cellular microenvironment using natural products was demonstrated to be able to alter inflammatory responses, favouring the regenerative pathways. This study also opens the way to a full investigation of RBE and its single components for the development of an effective formulation able to improve wound healing in oral and extra-oral tissues or to enhance in-vitro stem cell properties as tools for engineering graft applications.

## Additional file

Additional file 1: **Figure S1.** The effects of TNF-α on GMSCs and *Ribes nigrum* modulation of the inflammatory cytokine activity. A–D) GMSCs were treated in growth medium with different concentrations of TNF-α (1 ng/mL to 100 ng/mL) in the absence or presence of *Ribes nigrum* bud extract (RBE; 50 μg/mL) for 48 h (A, C) or 72 h (B, D). At the end of the treatments, the live cells were quantified using the neutral red assay, as described in the Methods. The data are expressed as a percentage with respect to the untreated cells (CTRL), which was set to 100%, and are presented as the mean values ± SEM of three independent experiments, each performed in duplicate. The significance of the differences was determined by one-way ANOVA, followed by Bonferroni’s post-hoc test or student *t* test: *P ≤ 0.05, **P ≤ 0.01 vs. the control; ^#^*P* ≤ 0.05, ^##^*P* ≤ 0.01 vs. the respective TNF-α. **Figure S2.** Apoptotic effects of TNF-α. GMSCs (A, B) and HMEC cells (C, D) were treated for 72 h; at the end, cells were collected, and the amount of phosphatidylserine externalization was evaluated using the Annexin V staining protocol. Representative plots of control (CTRL; A, C) and TNF-α 100 ng/mL (B, D) are presented. (DOCX 206 kb)

## Abbreviations

COX: Cyclooxygenase
ECM: Extracellular matrix
FBS: Fetal bovine serum
GMSC: Gingiva-derived mesenchymal stem cell
IL: Interleukin
MSC: Mesenchymal stem cell
PBS: Phosphate-buffered saline
RBE: *Ribes nigrum* bud extract
TGF: Transforming growth factor
TNF: Tumour necrosis factor
