# Supplementary material for: Negative effects of a high tumour necrosis factor-α concentration on human gingival mesenchymal stem cell trophism: the use of natural compounds as modulatory agents
Source: Stem Cell Res Ther. 2018 May 11;9:135. doi: 10.1186/s13287-018-0880-7 (PMC5948671; doi:10.1186/s13287-018-0880-7)
Supplement: Supplementary file 1 — Figure S1. The effects of TNF-α on GMSCs and Ribes nigrum modulation of the inflammatory cytokine activity. A–D) GMSCs were treated in growth medium with different concentrations of TNF-α (1 ng/mL to 100 ng/mL) in the absence or presence of Ribes nigrum bud extract (RBE; 50 μg/mL) for 48 h (A, C) or 72 h (B, D). At the end of the treatments, the live cells were quantified using the neutral red assay, as described in the Methods. The data are expressed as a percentage with respect to the untreated cells (CTRL), which was set to 100%, and are presented as the mean values ± SEM of three independent experiments, each performed in duplicate. The significance of the differences was determined by one-way ANOVA, followed by Bonferroni’s post-hoc test or student t test: *P ≤ 0.05, **P ≤ 0.01 vs. the control; #P ≤ 0.05, ##P ≤ 0.01 vs. the respective TNF-α. Figure S2. Apoptotic effects of TNF-α. GMSCs (A, B) and HMEC cells (C, D) were treated for 72 h; at the end, cells were collected, and the amount of phosphatidylserine externalization was evaluated using the Annexin V staining protocol. Representative plots of control (CTRL; A, C) and TNF-α 100 ng/mL (B, D) are presented. (DOCX 206 kb) [file 13287_2018_880_MOESM1_ESM.docx]

# Additional file 1

**Figure S1**

**Figure S1.** TNF-α effects on GMSCs and *Ribes nigrum* modulation of the inflammatory cytokine activity. A-D) GMSCs were treated in growth medium with different concentrations of TNF-α (1 ng/ml -100 ng/mL) in the absence or the presence of *Ribes nigrum* bud extract (RBE 50 µg/mL) for 48 h (A,C) or 72 h (B,D). At the end of the treatments, the live cells were quantified using the Neutral Red assay, as described in the Materials and Methods. The data were expressed as a percentage with respect to the untreated cells (CTRL), which was set to 100%, and they were presented as the mean values ± SEM of three independent experiments, each performed in duplicate. The significance of the differences was determined by one-way ANOVA, followed by Bonferroni’s post hoc test or student *t*-test: *P ≤ 0.05, **P ≤ 0.01 vs. the CTRL; # P ≤ 0.05, ## P ≤ 0.01 vs. the respective TNF-α.

**Figure S2**


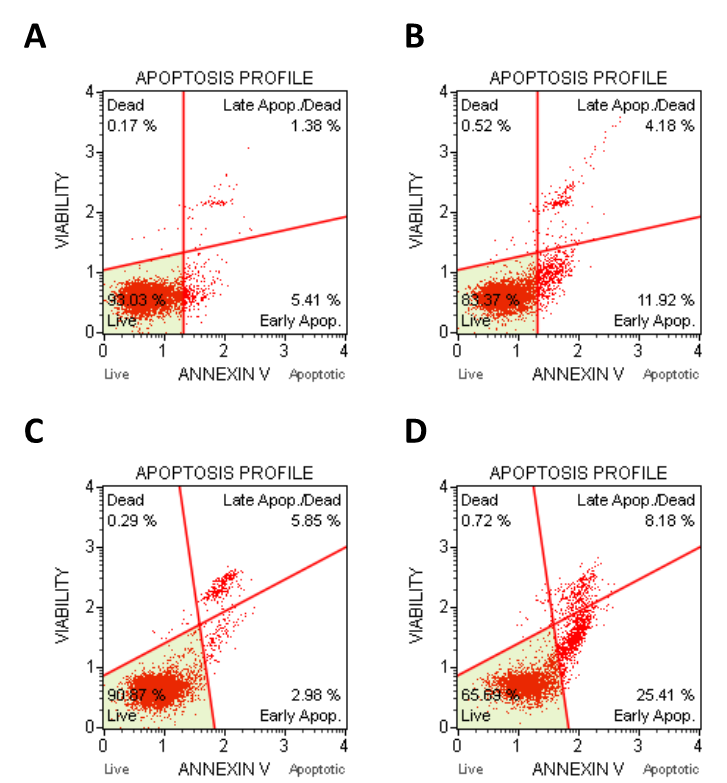


**Figure S2.** Apototic effects of TNF-α. GMSCs (A,B) and HMEC cells (C,D) were treated for 72h; at the end, cells were collected and the amount of phosphatidylserine externalization was evaluated using the Annexin V staining protocol. Representative plots of CTRL (A, C) and TNF-α 100 ng/ml (B,D) are presented.
